# Uniparental Disomy Reveals Hidden Genetic Causes of Congenital Heart Disease

**DOI:** 10.21203/rs.3.rs-10233423/v1

**Published:** 2026-07-20

**Authors:** Nahyun Kong, Javier Abello, Christopher Jongsoo Yoon, Andrew Ruttenberg, Zefan Li, Jahmiera Richee, Sarah Colijn, Kevin M. Bowling, Matheus Vernet Machado Bressan Wilke, Weilai Dong, Kenneth Ng, Elvisa Mehinovic, Purva Patel, Steven R. DePalma, Yung-Chun Wang, Tharaka Darshana Wijerathne, Jerome J. Lacroix, Yonghui Zhao, Rajan Sah, Jamison L. Nourse, Medha Pathak, Jessica Ensing, Stephanie Grainger, H Joseph Yost, Martin Tristani-Firouzi, Michael Wagner, Nicholas J. Ollberding, Jonathan Seidman, Sarah U. Morton, Bruce D. Gelb, Wendy K. Chung, Richard P. Lifton, Yidan Sun, John R. Edwards, Susan Dutcher, Christine E. Seidman, Obi L. Griffith, Malachi Griffith, Tim Schedl, Monkol Lek, Martina Brueckner, Amber N. Stratman, Sheng Chih Jin

**Affiliations:** 1 Department of Genetics, Washington University School of Medicine, St. Louis, MO, USA; 2 Department of Cell Biology and Physiology, Washington University School of Medicine, St. Louis, MO, USA; 3 Department of Medicine, Washington University School of Medicine, St. Louis, MO, USA; 4 Department of Genetics, Yale University School of Medicine, New Haven, CT, USA; 5 Laboratory of Human Genetics and Genomics, The Rockefeller University, New York, NY, USA; 6 Department of Pathology and Immunology, Washington University School of Medicine, St. Louis, MO, USA; 7 Department of Genetics, Harvard Medical School, Boston, MA, USA; 8 Department of Biomedical Sciences, Western University of Health Sciences, Pomona, CA; 9 Department of Cardiology, Washington University School of Medicine, St. Louis, MO, USA; 10 Department of Physiology and Biophysics, University of California, Irvine, CA; 11 Department of Biomedical Engineering, University of California, Irvine, CA; 12 Department of Cell Biology; Van Andel Research Institute, Grand Rapids, MI; 13 Senior Vice Provost for Research, The Catholic University of America, Washington, DC, USA; 14 Division of Pediatric Cardiology, University of Utah, Salt Lake City, UT, USA; 15 Divisions of Biomedical Informatics and of Biostatistics and Epidemiology, Cincinnati Children’s Hospital Medical Center, Cincinnati, OH, USA; 16 Division of Newborn Medicine, Department of Pediatrics, Boston Children’s Hospital, Boston MA, USA; 17 Department of Pediatrics, Harvard Medical School, Boston MA, USA; 18 Mindich Child Health and Development Institute and Department of Pediatrics, Icahn School of Medicine at Mount Sinai, New York, NY, USA; 19 Department of Pediatrics, Boston Children’s Hospital, Harvard Medical School, Boston, MA, USA; 20 Departments of Pediatrics and Medicine, Columbia University Medical Center, New York, NY, USA; 21 Center for Translational Bioinformatics, Washington University School of Medicine, St. Louis, MO, USA; 22 Center for Pharmacogenomics, Department of Medicine, Washington University School of Medicine, St. Louis, MO, USA; 23 Cardiovascular Division, Brigham and Women’s Hospital, Boston, MA, USA; 24 Howard Hughes Medical Institute, Chevy Chase, MD, USA; 25 Department of Pediatrics, Yale School of Medicine, New Haven, CT, USA; 26 Department of Pediatrics, Washington University School of Medicine, St. Louis, MO, USA

## Abstract

Congenital heart disease (CHD) affects ~1% of live births, yet the genetic basis of many cases remains unresolved. Uniparental disomy (UPD), the inheritance of both homologous chromosomes from one parent, is often overlooked. We developed TrioMix-UPD, an integrated short- and long-read sequencing framework for UPD detection and classification. Applying it to 3,740 CHD trios, we identified 12 UPD events, representing a 6.57-fold enrichment relative to the general population. Both advanced maternal age and enrichment of rare inherited variants in synaptonemal complex genes implicated meiotic chromosome segregation defects in UPD risk. Within UPD regions, we identified pathogenic homozygous variants in *PIEZO1* and *GLYR1* and nominate *MESD* as a novel CHD candidate gene. Functional studies in zebrafish and human cells recapitulated patient-specific cardiac phenotypes. Differential methylation analyses implicated imprinting dysregulation, including at the Prader-Willi critical region. Collectively, these findings establish UPD as an underrecognized contributor to CHD.

## INTRODUCTION

Congenital heart disease (CHD) is among the most common birth defects, affecting 1–1.8% of live births.^1^ Recurrence in families, twin concordance, and segregation studies underscore a substantial genetic contribution.^2,3^ A range of established etiologies that include chromosomal anomalies such as Trisomy 13, 18, 21, and monosomy X, as well as pathogenic inherited or *de novo* variants account for a subset of cases.^4–8^ Yet roughly 55% of CHD patients present without these associations and exhibit complex, non-Mendelian inheritance patterns, suggesting additional, less common genetic mechanisms contribute to CHD.

Uniparental disomy (UPD) represents one possible mechanism. In UPD, both copies of a chromosome originate from a single parent, typically arising from meiotic nondisjunction followed by mitotic rescue. UPD can occur as isodisomy, when two identical sister chromatids are inherited; heterodisomy, when both homologous chromosomes from one parent are transmitted; or mixed and segmental forms that arise through meiotic recombination or post-zygotic DNA repair (**Supplementary Figure 1**).^9^ By generating homozygous genotypes in isodisomic segments or altering the dosage of imprinted genes in the heterodisomic segments, UPD can generate profound phenotypic consequences.^10–12^ Although UPD is estimated to occur in only 0.05% of live births in the general population^13^, recent genomic studies have increasingly linked it to developmental and neurological disorders.^14–17^ However, despite growing recognition of UPD in clinical genomic testing laboratories, its role in CHD remains poorly characterized. Published reports are largely limited to isolated case studies of CHD identified in patients with an imprinting disorder or broader chromosomal syndromes involving chromosomes 4, 8, 9, 14, 15, and 16.^18–20^

**Figure 1.**
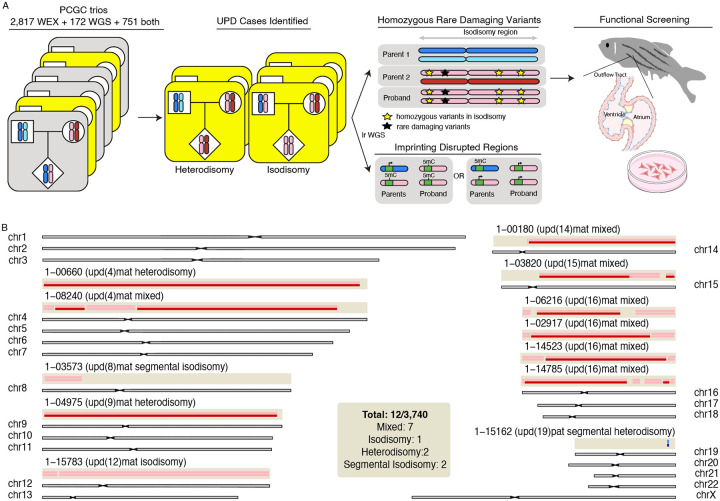
Study workflow and features of UPD events in the PCGC cohort. (A) Overview of the analytic pipeline. Whole-exome and/or whole-genome sequencing data from 3,740 PCGC parent–proband trios were screened for deviations from Mendelian inheritance using TrioMix-UPD to identify UPD. Within isodisomic segments, candidate variants were prioritized by identifying rare, likely pathogenic homozygous variants in the proband that were heterozygous in the UPD-contributing parent and absent in the other parent. Selected trios (n = 4) underwent PacBio HiFi sequencing to determine UPD breakpoints, identify structural variants, and assess methylation patterns. Top candidate variants were functionally evaluated in zebrafish. (B) Genomic locations of the 12 UPD events across chromosomes. Green bars denote isodisomy and purple bars denote heterodisomy (shown in chromosomal coordinates). (C) Maternal age at delivery for the full PCGC cohort, PCGC UPD cases, PCGC non-UPD cases, and 23andMe controls stratified by UPD status (boxplots show median, interquartile range, and whiskers).

The Pediatric Cardiac Genomic Consortium (PCGC) previously reported eight CHD probands with suspected UPD using an algorithm based on clustering of Mendelian errors^20^; however, no further in-depth evaluation was conducted. Here, we performed a comprehensive genomic analysis of an expanded PCGC cohort comprising 3,740 CHD trios using TrioMix-UPD, an enhanced version of TrioMix^21^ optimized for UPD detection across Illumina short-read and PacBio long-read sequencing. This updated algorithm enables UPD classification and breakpoint mapping. Using this approach, we systematically characterized UPD prevalence and chromosomal hotspots, investigated risk factors including maternal age and inherited variants in chromosome segregation genes. We further examined two mechanisms by which UPD may contribute to CHD: unmasking of pathogenic recessive variants and disruption of genomic imprinting, the latter assessed through DNA methylation profiling using METAFORA^22^. Functional studies in zebrafish and human cells provided validation of candidate genes (Figure 1A). Collectively, these results provide the first rigorous evaluation of UPD in CHD and establish a framework for the incorporation of UPD screening into routine genomic diagnostics for CHD and other rare disorders.

## RESULTS

### UPD is significantly enriched in CHD cases with hotspots on chromosomes 4 and 16

We analyzed genomic DNA from 3,740 parent-offspring trios enrolled through the PCGC, which is comprised of 2,817 probands with exome data, 172 with genome data, and 751 with both (Methods and **Supplementary Table 1**). Participants were recruited and consented in the United States and the United Kingdom based on a confirmed diagnosis of structural CHD. At the time of enrollment, comprehensive clinical assessments were conducted, including detailed cardiac findings, extracardiac anomalies (EC), and neurodevelopmental disorders (NDD; developmental delay, learning disability, intellectual disability, or autism diagnosed at ≥ 1 year of age (Methods and **Supplementary Table 1**).

To identify UPDs, we analyzed exome and genome sequencing data using TrioMix-UPD, an updated version of our previously published algorithm, TrioMix. We extended TrioMix^21^, which infers inheritance from variant-allele frequencies in trios with a newly implemented UPD detection mode that automates identification, subtype classification (isodisomy, heterodisomy, and mixed), and precise breakpoint mapping (Methods). Among 751 trios with both exome and genome data, UPD calls were cross validated within the same chromosome, and all calls were confirmed. We then compared UPD prevalence against the 23andMe cohort, comprising 214,915 parent–child trios and 916,712 parent-child duos genotyped by SNP microarrays.^13^

Among 3,740 PCGC trios, we identified twelve UPD events, none of which carried a previously reported pathogenic variant that explains the CHD phenotypes.^5–7^ Ten involved whole-chromosome UPD and two were segmental, corresponding to a prevalence of 0.32% (Table 1). When compared with 214,915 parent–child trios genotyped by 23andMe, in which 105 UPD cases observed (prevalence ~0.05%),^13^ the PCGC cohort showed a 6.57-fold enrichment of UPD (two-sample test for equality of proportions without continuity correction, *p* = 9.95×10^−13^).

We observed four maternal UPD events on chromosome 16, two on chromosome 4, and single maternal events on chromosomes 8, 9, 12, 14, and 15, as well as one paternal UPD on chromosome 19 (Figure 1B). Chromosome 16 exhibited the strongest enrichment, with 4 full-chromosome UPDs in 3,740 CHD probands versus 39 in 916,712 23andMe controls (odds ratio [OR] = 25; Fisher’s exact *p* = 7.1×10^−4^; per-chromosome analysis based on duos. The 23andMe report does not indicate which UPD events occurred in trios;^13^ **Supplementary Table 2**). A previously reported case of truncus arteriosus carrying isodisomy supports chromosome 16 as a recurrent hotspot for UPD-mediated CHD risk.^19^

### Predominant maternal origin and association with advanced maternal age

**Figure 2.**
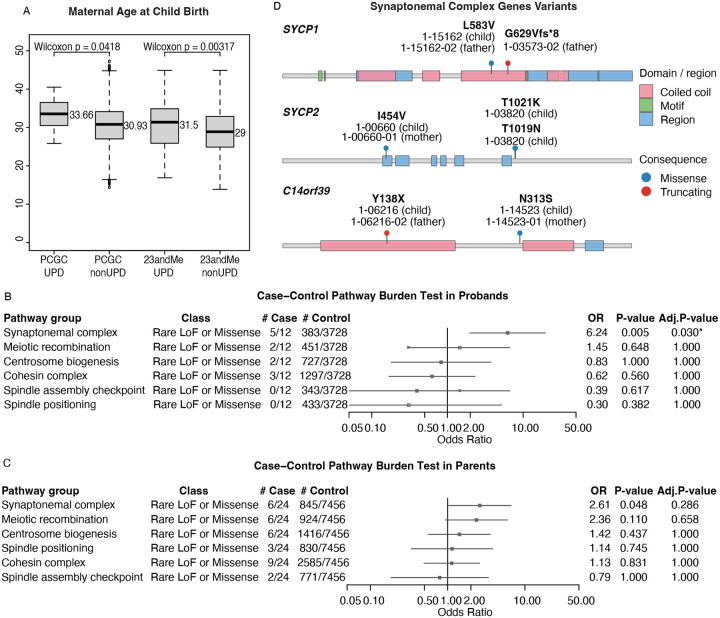
Maternal age and chromosome segregation pathway variant burden in uniparental disomy. **(A)** Maternal age at child’s birth by cohort and UPD status (PCGC and 23andMe); Data for the 23andMe cohort were obtained from Nakka et al. (2019). Boxplots show median, IQR, and 1.5× IQR whiskers. **(B)** Case-control pathway burden analysis in children. Rare (AF < 0.001) loss-of-function (LoF) or missense variants in genes from predefined chromosomal segregation pathways were aggregated and compared between cases and controls using Fisher’s exact test. Forest plots show odds ratios (ORs) with 95% confidence intervals (CIs), raw P values, and Bonferroni-adjusted P values. **(C)** Case-control pathway burden analysis in parents, performed as in (B). Forest plots show odds ratios (ORs) with 95% confidence intervals (CIs), raw P values, and Bonferroni-adjusted P values. **(D)** Lollipop plots of synaptonemal complex variants in *SYCP1*, *SYCP2*, and *C14orf39*, colored by consequence (blue, missense; red, truncating) and labeled with amino-acid change and carrier sample IDs/roles.

Of the twelve UPD events detected in PCGC probands, eleven were maternally inherited (two-sided exact binomial test *p* = 6.3×10^−3^; Table 1), consistent with prior reports linking UPD to errors in maternal meiosis.^23–25^ Because advanced maternal age is a recognized risk factor for nondisjunction, we compared maternal ages between cohorts. Mothers of all PCGC probands were, on average, older at delivery than mothers in the 23andMe cohort^13^ (median age of 30.9 vs 29 years; one-sided Wilcoxon signed rank test *p* < 2.2×10^−16^). The age difference was more pronounced for mothers of PCGC UPD cases (median 33.7 years), which was significantly higher than mothers of non-UPD PCGC probands (30.9 years; one-sided Wilcoxon rank sum test *p* = 0.04; Figure 2A). A similar enrichment of advanced maternal age among UPD cases was observed in the 23andMe control cohort, supporting a broader association between maternal age and UPD risk^13^ (median age of 31.5 vs 29 years; one-sided Wilcoxon rank sum test *p* = 0.00317; Figure 2A). In contrast, paternal age did not differ significantly between UPD and non-UPD groups in either cohort (median age of 35.7 vs 32.5 years; one-sided Wilcoxon rank sum test *p* = 0.2824; **Supplementary Figure 2**). These findings implicate maternal age as a contributor to the increased prevalence of maternally transmitted UPD observed in CHD.^23,25^

### Enrichment of rare inherited variants in the synaptonemal complex pathway among UPD families

Because UPD most commonly arises through chromosomal nondisjunction followed by chromosome rescue events^26^, we next investigated whether UPD families were enriched for rare inherited variants in genes involved in chromosome maintenance and segregation. We curated targeted gene sets spanning pathways implicated in meiotic recombination^27^, synaptonemal complex assembly^28^, cohesin regulation^29^, spindle assembly checkpoint signaling^30^, centrosome biogenesis^31^, and spindle positioning^32^ (Methods and **Supplementary Table 3**). Rare coding variants were defined as low-frequency loss-of-function (LoF) or missense variants (maximum population allele frequency ≤ 0.1% across gnomAD exomes^33^, gnomAD genomes^33^, and BRAVO^34^). Pathway-level and gene-level burden analyses in these pre-defined pathways were then performed separately in probands and parents from UPD trios, using non-UPD PCGC probands and parents as the respective controls (Methods).

Among UPD probands, pathway-level burden analysis showed the strongest enrichment in the synaptonemal complex pathway (OR = 6.24, 95% CI 1.97–19.75, Bonferroni adjusted *P* = 0.030) (Figure 2B). Five out of twelve UPD probands (1–00660, 1–03820, 1–06216, 1–14523, 1–15162) carried rare heterozygous variants in subset of the canonical synaptonemal complex genes, namely *SYCP1*, *SYCP2*, and *C14orf39* (Figure 2D, **Supplementary Figure S3** and **Supplementary Table 4**).^28^ Notably, the observed UPD configurations were consistent with meiotic error mechanisms associated with the disruption of synaptonemal complex function (**Supplementary Figure 1**). Proband 1–00660 exhibited maternal heterodisomy and carried a maternally inherited *SYCP2* missense variant p.I454V, which is absent from gnomAD v2.0.1^33^ and affects an evolutionarily conserved residue (GERP^35^ = 5.97) despite mixed computational predictions (SIFT^36^ tolerated, PolyPhen^37^ benign, Combined Annotation Dependent Depletion [CADD]^39^ = 2.3), suggesting that it may be causal for meiosis I nondisjunction resulting from defective homolog pairing and synapsis. Given the established role of SYCP2 in homolog alignment during meiotic prophase I, this observation supports a model in which impaired homolog synapsis predisposes to meiosis I segregation failure and subsequent heterodisomic UPD formation. Moreover, gene-level analyses in probands identified the strongest burden signals in *C14orf39* (Odds ratio [OR] = 15.66; CI 3.34–73.45; Bonferroni adjusted *P* = 0.47), *RAD51C* (OR = 16.85; CI 1.22–25.94; Bonferroni adjusted *P* = 1.00), *PDS5A* (OR = 5.62; CI 2.08–136.78; Bonferroni adjusted *P* = 1.00), and *SYCP2* (OR = 3.83; CI 0.83–17.61; Bonferroni adjusted *P* = 1.00) (**Supplementary Figure 3A**). Although none reached genome-wide significance, likely due to the limited sample size, these convergent signals further implicate genes involved in meiotic recombination and chromosome cohesion.

In parental burden analyses, *WDR90* showed the strongest gene-level signal, with rare LoF or missense variants identified in five individuals from four UPD families: 1–04975-01, 1–06216-02, 1–08240-02, 1–14523-01, and 1–14523-02 (OR = 3.17, CI 1.18–8.51; Bonferroni adjusted *P* = 0.033; **Supplementary Figure 3B** and **Supplementary Table 4**). Additional parental variants were observed in genes involved in meiotic recombination and chromosome cohesion, including *PDS5B, SYCP1, SGO2, MLH3, and STAG2* (**Supplementary Table 4**). At the pathway level, parents from the UPD families showed moderate enrichment of variants in synaptonemal complex genes (OR = 2.61, CI 1.03–6.59 1.188.51, Bonferroni adjusted *P* = 0.29) and meiotic recombination genes (OR = 2.36, CI 0.93–5.95, Bonferroni adjusted *P* = 0.66) (Figure 2C).

Together, these findings suggest that inherited defects affecting meiotic chromosome pairing, recombination, and segregation machinery may increase susceptibility to UPD in concert with advanced maternal age.

### UPD unmasks rare homozygous variants associated with CHD

Having identified enrichment of rare inherited variants in meiotic chromosome segregation pathways among UPD families, we next investigated whether UPD events unmasked homozygous pathogenic variants that could contribute to CHD. We first analyzed short-read sequencing data, focusing on rare homozygous single nucleotide variants (SNVs) and small insertions/deletions (indels) within isodisomic regions. Within UPD isodisomy regions, we identified 33 potentially damaging homozygous variants that are heterozygous in the UPD-contributing parent and absent in the other parent (See Method and **Supplementary Table 5**). Among them, four were classified as variants of uncertain significance (VUS) according to ACMG/AMP variant classification guidelines and 11 were in genes of uncertain significance (GUS) (Table 2).^38^

To capture more complex forms of genetic variation, we generated PacBio HiFi WGS data for three trios selected based on recurrent UPD (probands: 1–02917, 1–06216, 1–14785 and one trio with a known imprinting disorder (proband 1–03820, Prader-Willi Syndrome) (Figure 1B, Table 1, and Methods). Across these four trios, we identified 194 homozygous structural variants (SV) inherited from a heterozygous parent via UPD and absent in the other parent. Among them, 30 SV (15%) were rare (minor allele frequency [MAF] ≤1%, corresponding to an expected homozygous frequency of ≤ 1%) in 450 samples from 1000 Genomes Project sequenced by Oxford Nanopore Technologies long-read sequencing.^39^ After ACMG-based annotations with AnnotSV,^40^ 14 SVs overlapped a gene or regulatory element, passed IGV inspection, and were therefore prioritized as candidate SV (**Supplementary Table 6**). Using our SNV and SV data, together with predicted functional effect, evolutionary conservation, and gene expression in the developing mouse heart^7^, we selected three candidates, *PIEZO1*, *MESD*, and *GLYR1*, for investigation of the potential functional role of in regulating CHD-related phentoypes.

### Homozygous deleterious *PIEZO1* variants are associated with aortic outflow tract defects

**Figure 3.**
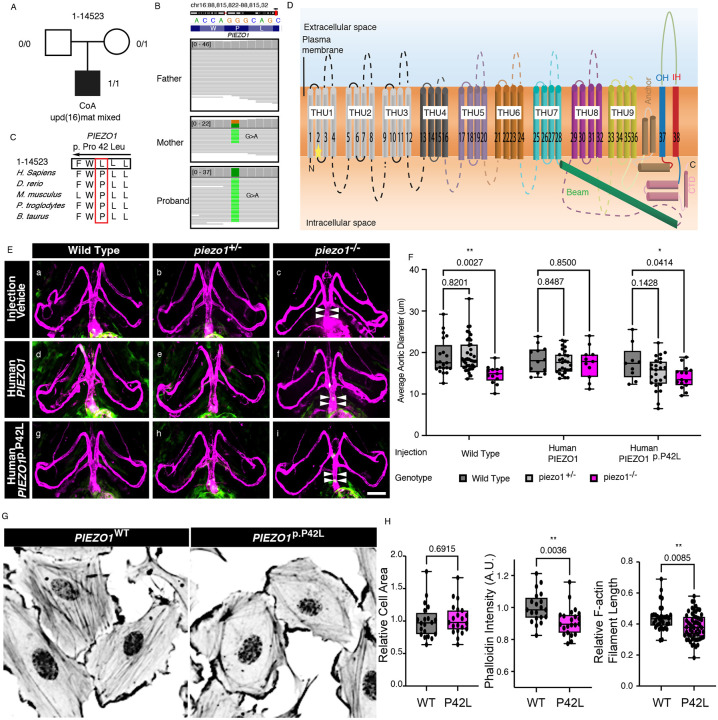
Rare homozygous *PIEZO1* missense variant unmasked by isodisomy and functional evaluation in zebrafish and HEK293T cell lines. (A) Trio pedigree for proband 1–14523 with coarctation of the aorta (CoA) and maternal UPD of chromosome 16. (B) Integrative Genomics Viewer (IGV) snapshot of *PIEZO1* p.Pro42Leu, showing the proband homozygous for the alternate allele, the mother heterozygous, and the father homozygous for the reference allele. (C) Multiple-sequence alignment demonstrating that Pro42 is highly conserved across species. (D) Predicted membrane topology of PIEZO1 (four-helix units; THU1–THU9) highlighting the position of Pro42 (yellow star). (*E*) Representative spinning-disk confocal images of the zebrafish cardiac outflow tract/aorta in wild-type, *piezo1*^+/−^, and *piezo1*^−/−^ embryos injected with vehicle, human *PIEZO1* (WT), or human *PIEZO1* p.Pro42Leu for rescue analysis. All embryos are *piezo2a.1*-null. Arrowheads indicate aortic narrowing consistent with coarctation. (Scale bar, 50 μm). (F) Quantification of aortic diameter across genotypes and rescue conditions. Each dot represents an individual zebrafish embryo. P-values were calculated using a two-way ANOVA with Sidak’s multiple comparisons test. Data are presented as box plots with min/max error bars. (G) Representative images of phalloidin immunostaining in human ECs (HUVECs) transfected with human *PIEZO1* WT or p.Pro42Leu plasmids. (H) Quantification of relative cell area, phalloidin staining intensity, and relative F-actin filament length. Each dot represents an individual cell, from 2 independent transfection and staining replicates. P-values were calculated using unpaired t tests. Data are presented as box plots with min/max error bars.

Among the homozygous variants identified within the UPD isodisomy regions, the most compelling candidate was *PIEZO1* p.Pro42Leu. *PIEZO1* encodes piezo-type mechanosensitive ion channel component 1. The p.Pro42Leu variant is heterozygous in the mother but homozygous in the proband (1–14523) who was diagnosed with coarctation of the aorta and had no extracardiac anomalies (Figure 3A–B). This variant is exceedingly rare in gnomAD v2.0.1 (MAF = 0, with no homozygotes reported), the proline is highly conserved across vertebrates, and the variant is predicted to be deleterious (CADD score = 26.7) (Figure 3C).^33,41^ PIEZO1 assembles as a trimeric, three-bladed mechanosensitive channel expressed in many cell types in the cardiovascular system including endothelial cells, cardiomyocytes, and blood cells, sensing shear stress from blood flow.^42–44^ Each subunit comprises 38 transmembrane (TM) helices organized into nine four-helix units and a central pore domain (Figure 3D). Although the structure of TM helices 1–12 remains unresolved experimentally, AlphaFold2 predicts that Pro42 lies at the C-terminus of TM2 (Figure 3D).^45^

To test the role of PIEZO1 in heart development we turned to zebrafish where *piezo* genes have established roles in development of the cardiac outflow-tract (OFT), the connection between the left ventricle and the great arteries.^46–48^ Unlike humans, who carry two PIEZO paralogs (*PIEZO1* and *PIEZO2*), zebrafish possess multiple *piezo* genes (*piezo1, piezo2a.1, piezo2a.2,* and *piezo2b*) due to genome duplication. To overcome paralog redundancy, all experiments were performed using a *piezo2a.1*^−/−^ mutant background, which does not independently exhibit OFT phenotypes (Figure 3E).^46–48^ For clarity and brevity, subsequent results focus exclusively on the *piezo1* genotype. *piezo1*^−/−^ homozygous mutants exhibited ~20% reduction in OFT aortic only diameter (Figure 3E. c white arrow heads). This narrowing was absent in *piezo1*^+/+^ wild-type and *piezo1*^+/−^ heterozygous conditions (Figure 3E. a–b).

To assess the functional impact of the human *PIEZO1* p.Pro42Leu variant on aorta and OFT development, we performed plasmid microinjection rescue experiments by re-expressing human wild-type *PIEZO1* or the human p.Pro42Leu *PIEZO1* variant in zebrafish *piezo1*^−/−^ mutants and their siblings. Introduction of human wild-type *PIEZO1* into *piezo1*^+/+^ and *piezo1*^+/−^ zebrafish did not affect OFT aortic diameter (Figure 3E. a,b,d,e); however, in *piezo1*^−/−^ mutants it restored aortic diameter to wild-type levels (Figure 3E. c,f). In contrast, the human *PIEZO1* p.Pro42Leu variant failed to restore the reduced OFT diameter observed in zebrafish *piezo1*^−/−^ mutants, suggesting that this variant is nonfunctional in this context (Figure 3E. i). The human *PIEZO1* p.Pro42Leu variant did not induce phenotypes in *piezo1*^+/+^ or *piezo1*^+/−^ siblings (Figure 3E. g,h), indicating that its pathogenicity is likely limited to its homozygous form and does not act as a dominant-negative protein. Based on the failure to rescue the phenotype in a non-human knockout model, these findings provide functional evidence supporting a loss-of-function mechanism and contribute to future assessment of gene–disease validity between PIEZO1 and CHD, in accordance with the ClinGen Gene Curation framework.^49^ In this framework, rescue experiments are scored based on model system evidence, where restoration of phenotype via the wild-type gene, but not the variant, supports a causal role for the gene in disease.

We next assessed the functional impact of the PIEZO1 p.Pro42Leu variant on channel activity using two assays. First, we measured whole-cell ionic currents in response to mechanical indentations from cells transiently transfected with a PIEZO1-GCaMP6f plasmid carrying or not the p.Pro42Leu mutation (**Supplementary Figure 4**).^44^ Electrophysiology recordings showed that, although both WT and p.Pro42Leu PIEZO1 channels produce ionic currents with similar inactivation time-constant (Tau) in response to indentations, p.Pro42Leu channels tended to produce lower current density compared to WT channels (**Supplementary Figure 4A**). We then performed Ca^2+^ imaging from HUVECs transfected with PIEZO1-GCaMP6f (WT) or p.P42L PIEZO1-GCaMP6f.^44^ Similarly, the p.Pro42Leu variant showed a decrease in the density of calcium-dependent fluorescence puncta compared to WT channels (as measured by GFP antibody staining following fixation, **Supplementary Figure 4B**). Taken together with the patch clamp data, this suggests that the main phenotypic consequence of the PIEZO1 p.Pro42Leu variant is likely reduced channel expression or mislocalization rather than an inability to activate when mechanically stimulated. Finally, expression of the p.Pro42Leu variant led to disrupted F-actin organization in transfected cells, as measured by phalloidin staining intensity and actin fiber lengths (Figure 3G–H), suggesting that expression of this variant can elicit changes in cellular architecture. Collectively, our findings implicate that UPD-generated unmasking of homozygous *PIEZO1* p.Pro42Leu results in functional but reduced channel expression, leading to aortic outflow-tract narrowing possibly via impaired actin stability.

Beyond the UPD trios, we identified five additional non-UPD CHD probands carrying rare (MAF ≤ 10^−3^) deleterious (LoF or missense variants with CADD ≥ 10 or MetaSVM deleterious) *PIEZO1* variants within the 3,740 PCGC cohort. Specifically, these included one homozygous p.Tyr1264Cys from 1–00674, as well as four compound heterozygous genotypes consisting of p.Met711Lys with p.Phe994Cys from 1–02244, p.Lys2520Glu with p.Ser1346Ala from 1–03173, p.Pro1770Leu with a nonframeshift insertion p.Glu756delinsGluProPro from 1–13022, and p.Tyr582Cys with p.Ala2237Gly from 1–15783. These variants were distributed across multiple regions of *PIEZO1*. Four of the five non-UPD probands with *PIEZO1* variants had pulmonary stenosis (pulmonary valvar stenosis with or without septal defects, tetralogy of Fallot with or without bicuspid aortic valve), and three of five had isolated CHD. The two probands with extracardiac anomalies included one participant with bilateral talipes and umbilical hernia, and another participant with unilateral renal agenesis who was also 47 XXX. Together, these observations support a broader role for rare deleterious *PIEZO1* variants in cardiovascular septation, aortic arch remodeling, and cardiac outflow tract development.

### An *MESD* homozygous variant associated with septal defects in humans

**Figure 4.**
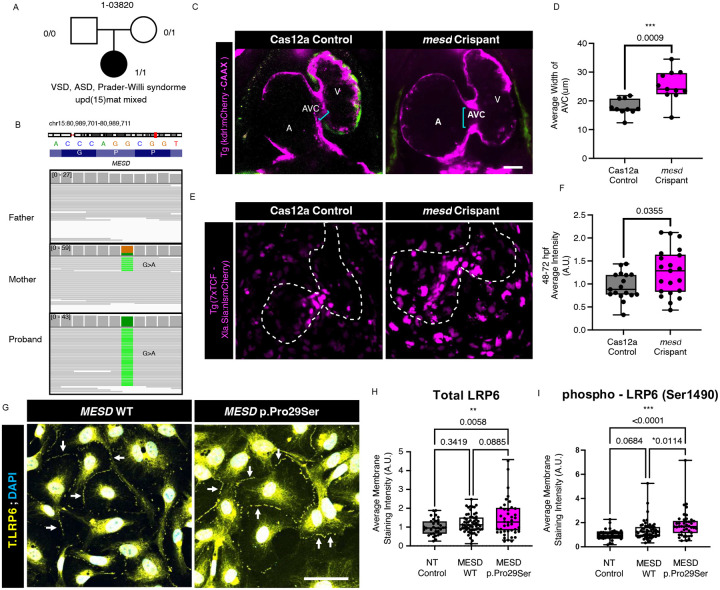
Rare homozygous deleterious *MESD* missense variant unmasked by isodisomy and linked to altered cardiac development and Wnt signaling in zebrafish and human umbilical vein endothelial cells (HUVECs). (A) Trio pedigree for proband 1–06216 with atrial septal defect (ASD) and maternal UPD of chromosome 16. (B) Multiple-sequence alignment showing that *MESD* Pro29 is highly conserved across species. (C) Spinning-disk confocal images of zebrafish hearts following G0 CRISPR/Cas12a targeting of *mesd* (*mesd* crispant) versus Cas12a-only control in the *Tg(kdrl:mCherry-CAAX)* reporter line. Representative hearts are shown (scale bar, 25 μm). A, atrium; V, ventricle; AVC, atrioventricular canal. (D) Quantification of average AVC width at 72 hpf in control and *mesd* crispant embryos. Each dot represents an individual zebrafish embryo. (E) Spinning-disk confocal images of *Tg(7xTCF-Xla.Sia:nlsmCherry)* embryos injected with *mesd* CRISPR or Cas12a-only control to assess Wnt/β-catenin reporter activity in 48–72 hpf hearts. White dashed lines outline the heart (72 hpf). (F) Quantification of Wnt reporter intensity, shown as the ratio of *mesd* CRISPR–injected signal relative to Cas12a control (arbitrary units, AU). Each dot represents an individual zebrafish embryo. P-values were calculated using unpaired t-tests for (D) and (F). Data are presented as box plots with min/max error bars. (G) Representative immunostaining images of total LRP6, in *MESD* WT and *MESD* Pro29Ser variant transfected HUVECs. Cells were fixed 24 hours after transfection of the indicated plasmid. Arrows highlight plasma membrane boundaries. (Scale bar, 50 μm). (H-I) Quantification of the total LRP6 and the phospho-LRP6 membrane labeling intensity per condition. Regions identified by phalloidin labeling at cell-cell boundaries were used for intensity measurement. Each dot represents an individual image, images from at least 3 independent experiments were analyzed. P-values were calculated using ordinary one-way ANOVAs with Tukey’s multiple comparisons test for (H) and for Kruskal-Wallis test with Dunn’s multiple comparisons test for (I). Data are presented as box plots with min/max error bars.

In the proband 1–03820, who presented with an ostium-primum atrial septal defect (ASD), VSD, and cleft mitral valve, we identified a homozygous p.Pro29Ser variant in *MESD*, a gene not previously associated with CHD (Figure 4A). The proband also had coloboma, microcephaly, growth hormone deficiency, precocious puberty, congenital scoliosis, and a dysplastic kidney.

This variant was heterozygous in the mother, absent in the father, and was extremely rare in gnomAD v2.0.1 (MAF = 3.23×10^−5^, with no homozygotes reported), (Figure 4B). *MESD* encodes a chaperone for low-density lipoprotein receptor-related protein (LRP),^50^ plays a critical role during mesoderm development, and is a known cause of recessive osteogenesis imperfecta.^51^ *mesd* G0 Crispant screening in the zebrafish identified that double-strand cutting of *mesd* leads to widening of the atrioventricular canal (AVC) and dilation of the overall heart structure (Figure 4C–D), this phenotype is often seen in zebrafish, which lack a four chambered heart, to track with human septal defects. In our 3,740 PCGC cohort, we identified one additional non-UPD proband (1–02806) harboring a rare (MAF =0.005 in gnomAD v.2.0.1), homozygous *MESD* variant (p.Ser4Phe) with a CADD score of 19.6, who presented with an atrial septal defect and abnormal branching pattern of the left aortic arch, with no extracardiac anomalies, further supporting a potential role for MESD in cardiovascular septation and vascular remodeling defects.

MESD is known to function as an LRP5/6 chaperone^50^ or as a Wnt inhibitor by blocking LRP5/6 binding of Wnt ligands.^50,52–54^ LRP6 activation (phosphorylation) occurs in response to Wnt ligand engagement with the receptor complex, and its stabilization and transport to the plasma membrane is regulated by MESD.^51,52^ Therefore, we examined Wnt/β-catenin signaling in the zebrafish using an established reporter line: *Tg(7xTCF-Xla.Sia:nlsmCherry)*^*ia5*^ (Figure 4E)^55^, and found an increase in Wnt reporter activity coupled with an expansion of Wnt positive cells to outside of the AVC at 72 hpf in *mesd* crispant embryos compared to Cas12a controls (Figure 4F). To evaluate how the human *MESD* p.Pro29Ser variant influences LRP6, we transfected human endothelial cells (HUVECs) with either wild-type human *MESD* or the p.Pro29Ser variant and immunostained for phosphorylated and total LRP6 protein (Figure 4G and **Supplementary Figure 5**). HUVECs expressing the *MESD* p.Pro29Ser variant showed increased plasma membrane levels of both phosphorylated and total LRP6 (Figure 4H–I), consistent with an increase in Wnt signaling activity in response to this *MESD* variant. Together, these zebrafish and cellular assays suggest MESD as a plausible CHD-contributing gene, and that the *MESD* p.Pro29Ser variant enhances LRP6-dependent Wnt/β-catenin signaling.

### A variant in the *GLYR1* 3′-untranslated region (UTR) tandem repeat associated with ASD

**Figure 5.**
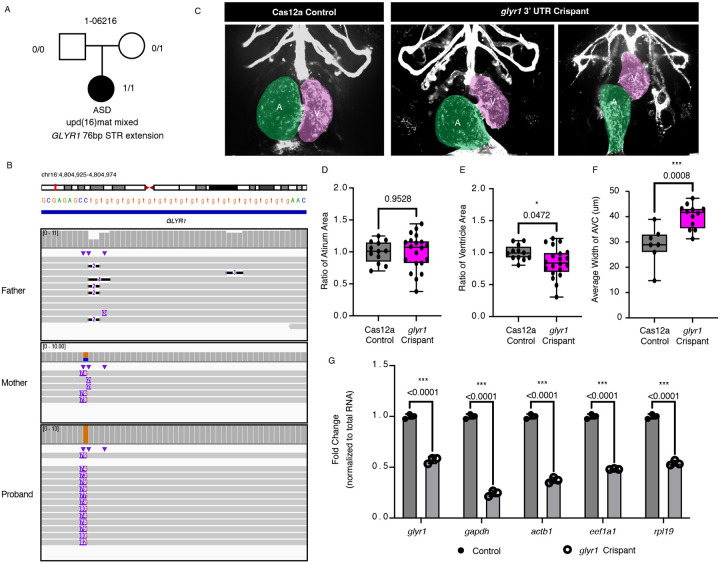
Homozygous *GLYR1* short tandem repeat (STR) expansion unmasked by isodisomy and functional evaluation in zebrafish. (A) Trio pedigree for proband 1–06216 showing a homozygous *GLYR1* STR expansion within an isodisomy region. (B) Integrative Genomics Viewer (IGV) visualization of trio PacBio HiFi long-read data supporting the *GLYR1* STR expansion. (C) Spinning-disk confocal images of 72 hpf zebrafish hearts following CRISPR/Cas12a targeting of the *glyr1* exon 13/14 splice junction (*glyr1* crispants) versus Cas12a-only control in the *Tg(kdrl:mCherry-CAAX) line*. A, atrium (green); V, ventricle (magenta). Images are pseudocolored for visualization. (D–E) Quantification of atrium and ventricle chamber areas, shown as the ratio of *glyr1* crispants to Cas12a controls. (F) Average width of the atrioventricular canal (AVC). Statistics were done with a two-sided t-test (exact p values are shown). P-values were calculated using unpaired t-tests for panels D-F. Data are presented as box plots with min/max error bars. (G) qPCR analysis of Cas12a Control versus *glyr1* Crispants, demonstrating decreased mRNA transcript levels present in animals with disruptions to the exon 13/14 splice junction of the *glyr1* gene. P-values were calculated using a two-way ANOVA with Sidak’s multiple comparisons test. Data are presented as the mean ± SD.

In a patient 1–06216, who presented with ASD, long-read sequencing revealed a biallelic 76-bp TG tandem repeat in the 3′ UTR of the Glyoxylate Reductase 1 Homolog (*GLYR1*) gene (Figure 5A–B). This repeat was absent in the father and heterozygous in the mother. *GLYR1* encodes a chromatin reader that is involved in transcription initiation and elongation via chromatin remodeling, and has been shown to be highly expressed in the embryonic heart (E14.5, 93rd percentile) and co-regulate cardiac developmental genes.^56^ Prior studies have linked dominant *de novo* and frameshift *GLYR1* variants to CHD, supporting its identification as a candidate gene.^56,57^ As *GLYR1* has a pLI=1^58^, indicating that the gene is loss-of-function intolerant, the 76-bp TG tandem repeat may result in a weak hypomorphic effect. The heterozygous mother does not have a reported CHD. Functional experiments in zebrafish showed that introduction of double stranded breaks at the exon 13/14 splice junction of *glyr1,* using CRISPR/Cas12a G0 genome editing, leads to malformations of the heart, including decreased ventricle area, laterality defects, and dilation of the AVC (Figure 5C–F). Exon 14 is the small, terminal exon of *glyr1*, immediately adjacent to the 3` UTR and disruptions at this site are predicted to lead to instability of the region (**Supplementary Figure 6B**). qRT-PCR analysis of Cas12a control-injected embryos compared to *glyr1* Crispant embryos demonstrated a marked decrease in mRNA levels across a number of target genes (Figure 5G). This decrease in mRNA levels is consistent with the known functions of *glyr1* and the notion that variants that disrupt the C-terminal end of this gene can be causative of developmental malformations. While not identical to the 3` UTR tandem repeat identified in the proband, these studies support a role for mutations at the 3` end of *glyr1* in regulating heart formation and suggest follow-up studies are warranted.

### Long-read sequencing identified Prader-Willi locus imprinting and novel UPD-associated imprinted loci in CHD

Because UPD can contribute to disease through imprinting-related dysregulation, we profiled DNA methylation using PacBio long-read sequencing in four proband-parent trios and 82 additional unaffected parents from PCGC. CpG methylation levels were quantified for each CpG site across the four probands and all 90 parents. To identify regions in which a proband showed contiguous deviation from the parental reference distribution, we applied METAFORA^22^, which computes the absolute methylation difference relative to the population median and an absolute methylation z-score (see Methods).

**Figure 6.**
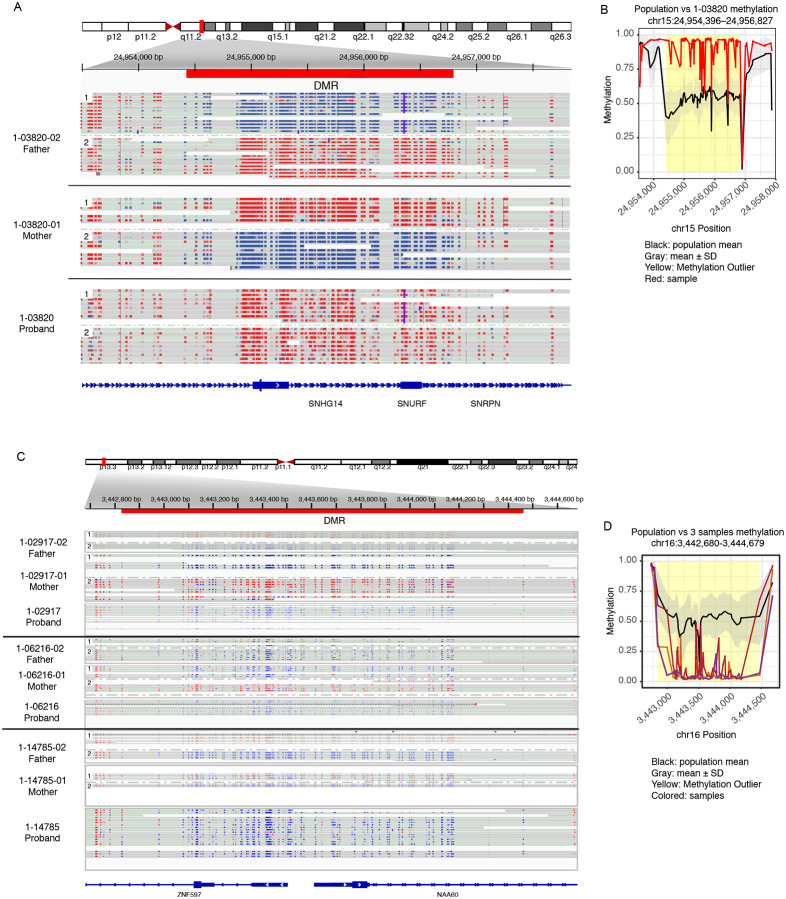
UPD-associated imprinting effects revealed by long-read sequencing (A) IGV screenshot of methylation outlier region detected in a Prader-Willi syndrome (PWS) patient with parents. DMR; Differential Methylated Region. (B) Methylation level of the methylation outlier region in the 90 healthy parents’ population and the PWS patient. (C) IGV screenshot of the methylation outlier region detected in three chr16 UPD patients with their parents. (D) Methylation level of the methylation outlier region in the 90 healthy parents’ population and three chr16 UPD patients.

Across the four probands, we identified eleven methylation outliers (z-score > 2). To assess whether these methylation abnormalities could be explained by defects in core DNA methylation machinery, we examined rare coding variants in genes involved in DNA methylation establishment and maintenance, including *DNMT3A*, *DNMT3B*, *DNMT3L*, *DNMT1*, *TET1*, *TET2*, and *TET3*. We did not identify any rare nonsynonymous or predicted loss-of-function variants in these genes in any of the four probands. Notably, proband 1–03820, who carries maternal UPD of chromosome 15 and the CHD-associated *MESD* p.Pro29Ser homozygous variant (see above), showed hyper-methylation at the SNURF–SNRPN imprinting control region (chr15: 24,954,396 – 24,956,827; Table 3 and Figure 6 A–B). In contrast, the population shows a mean methylation level of 53%, whereas the proband’s parents display the expected allele-specific differential methylation pattern. This region normally exhibits parent-of-origin–specific methylation that maintains expression of paternally expressed genes across the Prader–Willi locus.^59^ In maternal UPD15, the absence of the paternal allele results in an abnormal methylation configuration and silencing of the paternal gene program, a hallmark molecular mechanism underlying Prader–Willi syndrome (PWS), consistent with this patient’s clinical diagnosis. Interestingly, in this proband, we also detected a hypermethylated interval (chr15:24,477,570–24,478,450) within the annotated ncRNA locus *LOC105370733* in the Prader–Willi critical region (15q11.2–q13). Notably, a nearby ncRNA, *PWRN1*, has been reported to show imprinted expression in the fetal brain and was inferred to be implicated in the establishment of paternal imprinting within the Prader–Willi locus.^60^ Although *LOC105370733* remains poorly characterized, it may also have imprinted expression, and disruption of its monoallelic methylation could contribute to PWS pathogenesis.

In addition to the chr15 imprinting defect, we observed methylation disruptions at established imprinting loci, including *NAA60* (proband 1–12917) and *ZNF597* (probands 1–12917 and 1–14785) (Table 3 and Figure 6C–D). NAA60 encodes a Golgi-associated N-terminal acetyltransferase involved in post-translational acetylation of membrane proteins and maintenance of Golgi integrity,^61^ whereas *ZNF597* encodes a KRAB zinc-finger transcriptional regulator located within a maternally expressed imprinted locus.^62^ Interestingly, *NAA60* and *ZNF597* are expressed in the developing mouse heart at embryonic day 14.5, with corresponding expression percentile ranks of 68.5 and 43.1, respectively.^63^ Collectively, these analyses nominate additional candidate imprinted loci whose altered methylation profiles may perturb gene regulation and contribute to UPD-associated CHD, although further functional studies will be required to establish causal mechanisms.

## DISCUSSION

This study provides, to our knowledge, the first to systematically investigate the impact of all types of UPD — including isodisomy, heterodisomy, mixed, and segmental UPD — in CHD. Leveraging the largest trio-based CHD cohort to date, together with long-read sequencing and functional modeling in zebrafish and human cells, we were able to overcome several limitations of previous methods based on SNP microarrays^13,16^ or short-read sequencing^15,64,65^, which often lacked sufficient resolution to accurately define UPD structure, breakpoints, or methylation patterns. The complete parent–offspring trio design of the PCGC cohort further enabled comprehensive detection and classification of all UPD subtypes, including forms that are difficult to resolve in incomplete family structures.^14,16,17^

Within the expanded PCGC cohort, we recovered all eight suspected UPD events previously reported^20^ and identify four new UPD events. We utilized a uniformly processed cohort and a novel UPD-calling framework that detects deviations from Mendelian inheritance and can identify both isodisomy and heterodisomy with high accuracy.^21^ We followed this with PacBio long-read sequencing and evaluated several putative homozygous variants and functionally modeled three of them using *in vitro*/*in vivo* models. Together, these data support that UPD can unmask damaging recessive variation and suggest one plausible mechanism by which UPD events may contribute to CHD risk. This detailed genomic approach, combining short-read sequencing detection, long-read sequencing investigation, and functional characterization using the zebrafish and cell-based models, provides a framework for gene discovery centered on UPD in Mendelian disorders.

We observed a strong association between advanced maternal age and maternally derived UPD, corresponding to an approximately six-fold enrichment in UPD events within the PCGC cohort compared with the 23andMe general population cohort^13^. Mechanistically, UPD most commonly arises from maternal meiotic nondisjunction, a process known to increase with age, providing a plausible biological basis for this association.^23–25^ The higher average maternal age across the PCGC relative to the 23andMe general population controls may also suggest an independent relationship between maternal age and CHD risk, although disentangling maternal-age effects from UPD-mediated risk will require additional study.

Beyond maternal age, our burden analyses further suggest that inherited rare variation in meiotic chromosome segregation pathways may predispose families to UPD formation. We observed enrichment of rare variants affecting genes involved in synapsis and meiotic recombination among UPD trios, supporting a model in which UPD may arise not only from stochastic nondisjunction events, but also through inherited perturbations altering meiotic chromosome biology.

Several families illustrated mechanistically coherent relationships between UPD subtype and the biological functions of inherited variants. In family 1–00660, which carried maternal heterodisomy of chromosome 4, the mother as well as the proband shared a rare *SYCP2* missense variant. Because *SYCP2* encodes a core component of the synaptonemal complex required for homolog pairing and synapsis during meiosis I, impaired synapsis provides a plausible mechanism for homolog segregation failure leading to heterodisomy. Notably, *SYCP2* has a pLI score of 1.00, indicating that it is highly intolerant to heterozygous loss-of-function variation, further supporting the functional relevance of the inherited variant identified in this family. Additionally, family 1–15783, which exhibited maternal isodisomy of chromosome 12, carried a maternally inherited *STAG3* variant. *STAG3* encodes a meiosis-specific cohesin subunit essential for sister chromatid cohesion, and cohesin dysfunction is a well-established cause of meiosis II chromosome missegregation, consistent with the expected mechanism underlying isodisomy.^66^

Interestingly, we found rare LoF or missense variants from five UPD parents (1–04975-01, 1–06216-02, 1–08240-02, 1–14523-01, and 1–14523-02). *WDR90* encodes a centrosome-associated WD-repeat protein involved in centriole architecture and microtubule organization, and has been implicated in centrosome biology.^67^ Because centrosome integrity could be linked to faithful chromosome segregation via spindle organization, variation in *WDR90* provides a biologically plausible link to UPD-generating nondisjunction events. However, both STAG3 and WDR90 have low pLI scores^58^ (pLI = 0 for both), suggesting tolerance to heterozygous loss-of-function variation at the population level; the precise genetic mechanisms by which variants in these genes may contribute to UPD in these families therefore remain to be determined.

Notably, several potentially contributory variants were inherited from fathers, including the paternal *SYCP2* variant in family 1–14523 and a paternal *DYNC1H1* variant in family 1–15783. *DYNC1H1* encodes the heavy chain of cytoplasmic dynein, which is required for spindle organization and chromosome movement during cell division.^68^ Although discussions of UPD and aneuploidy have historically focused primarily on maternal meiotic errors, these observations raise the possibility that paternal defects in chromosome segregation pathways may also contribute to UPD formation. One potential mechanism is gamete restoration (also termed gamete complementation), in which a disomic gamete from one parent fertilizes a nullisomic gamete from the other, resulting in the inheritance of both homologs from a single parent.^69^ Under this model, paternal segregation defects could contribute indirectly to maternal UPD formation by increasing the probability of paternal nullisomic or disomic gametes. While these observations remain correlative and require functional validation, the convergence of UPD subtype, gene function, parent-of-origin, and predicted meiotic mechanism across multiple independent families provides a coherent framework linking inherited chromosome segregation defects to UPD susceptibility.

UPD is often enriched in disease cohorts because it can increase disease risk through multiple mechanisms.^15,64^ For example, isodisomic UPD can convert a heterozygous pathogenic variant on one parental haplotype into homozygosity in the proband, thereby unmasking recessive alleles. This recessive mechanism is expected to be particularly consequential when the UPD segment includes genes that are markedly depleted for homozygous variation in the general population.^33^ Importantly, UPD does not alter copy number and change dosage. Pathogenic effects are likely driven by changes in imprinting-related gene expression and/or homozygosity.

Because UPD events often span large genomic intervals (frequently >20 Mb) or even entire chromosomes, a single event affects many genes simultaneously and increases the likelihood of multiple clinically relevant consequences. For example, patient 1–03820 with chromosome 15 UPD developed PWS due to hypermethylation at the *SNURF–SNRPN* imprinting control region, and also had CHD associated with a homozygous *MESD* p.Pro29Ser variant. These findings underscore how single large-scale chromosomal abnormalities can lead to a combined clinical or more complex presentations.

We identified twelve UPD events in individuals with CHD and assessed the potential clinical relevance of detected homozygous variants. Using standard clinical variant interpretation criteria, 4 variants were classified as VUS, and 11 resided within GUS. For the VUS, the current evidence is insufficient to establish causality, most commonly due to limited gene–disease evidence, lack of recurrence in independent CHD cohorts, and the absence of functional data demonstrating a cardiac developmental effect. To strengthen interpretation, important next steps include functional validation of prioritized candidate variants, for example *in vivo* or *in vitro* cardiac models, and replication in additional CHD datasets. Additional support could come from identifying rare inherited variants in the same candidate genes among non-UPD PCGC patients and from orthogonal molecular evidence within the UPD segments, such as allele-specific expression or imprinting-related effects where relevant. We highlight prioritized candidate variants, including several rare, UPD-unmasked SVs overlapping genes or regulatory elements, as plausible contributors that warrant follow-up. Among the 54 SVs identified, recurrent events affecting *RAB11FIP3, CACNA1H, CDH13, ZNF469, ACSF3,* and *CPNE7* were observed across multiple samples, nominating these genes as putative CHD genes in our UPD patients (**Supplementary Table 6**). However, some UPD events may represent incidental findings or phenotypic modifiers rather than direct causes of disease.

In our study, we identified a variant unmasked by UPD in the *MESD* gene, which is known to function in WNT signaling, but has not previously been implicated in CHD. WNT-mediated signaling is a core developmental pathway that is precisely regulated in a stage-specific manner during cardiogenesis, providing biologically plausible role for MESD to link the identified loci to cardiac malformations. *MESD* encodes an endoplasmic reticulum chaperone required for proper folding and trafficking of LRPs, including the Wnt co-receptors LRP5/6, and has also been described as a functional antagonist of Wnt signaling through LRP binding in some contexts.^50^ Tight spatiotemporal regulation of canonical Wnt/β-catenin signaling is essential for heart development, with pathway misregulation known to disrupt cardiac progenitor specification, endocardial cushion formation, and AVC patterning—processes directly relevant to septation defects such as ASD/VSD.^70^ Consistent with this framework, our human cell and zebrafish data show that *MESD*/*mesd* disruption increases Wnt signaling activation within the AVC that is coupled with structural heart defects, supporting a model in which the *MESD* p.Pro29Ser variant perturbs LRP/Wnt homeostasis to impair cushion/valve development and heart septation. Currently, our studies cannot differentiate whether the increased signaling is derived from amplified stability and ER to plasma membrane transport of LRP6 due to the *MESD* variant, or inability of this variant to interact with the Wnt ligand binding pocket of LRP6 at the cell surface. Moving forward, this point will be interesting to distinguish with in-depth follow-up studies. Notably, in other contexts, recessive *MESD* variants have been linked to human disease by affecting mesoderm-derived tissues (e.g., recessive osteogenesis imperfecta), further supporting that biallelic *MESD* dysfunction can be pathogenic.^51^

Our work also identified and provides functional support for additional genes previously implicated in CHD pathogenesis, in particular *PIEZO1*. PIEZO1 has been intimately linked to proper cardiovascular development, formation of valves in both the heart and lymphatics, and even in the progression of thoracic aortic aneurysms in Marfan syndrome.^46,47,71–75^ Further, biallelic variants in *PIEZO1* have been reported in individuals with autosomal recessive lymphatic dysplasia, a disorder characterized by a high incidence of non-immune hydrops fetalis and childhood-onset of facial and four limb lymphoedema.^76,77^ Our proband was identified to harbor a *PIEZO1* p.Pro42Leu variant inherited via maternal UPD. The proline in this position is predicted to be at the C-terminal end of TM2 in the PIEZO1 blades. A proline at this site could be responsible for introducing ‘flexibility’ in the protein structure allowing for proper PIEZO1 blade curvature,^78^ with the leucine substitution in the proband possibly making TM2 less flexible and impairing channel stability and/or localization. While additional experimental data is needed to support this proposed mechanism, our results provide an interesting example of how a rare variant that alters protein structure can drive disease pathogenesis.

We found significant enrichment of chromosome 16 UPD in our CHD cohort. Maternal UPD16 often arises from a trisomic conceptus followed by trisomy rescue early in development, which may occur in a subset of cells to produce residual mosaicism.^79^ It has been argued that clinical features of maternal UPD16 are attributable to trisomy 16 mosaicism rather than UPD itself.^80^ While the clinical spectrum is highly variable, CHD is recurrently reported in mosaic trisomy 16.^81^ The marked phenotypic variability may reflect the timing and completeness of trisomy rescue and the tissue distribution of residual trisomic cells.^82^ Previously, tissue-restricted persistence of trisomy has been demonstrated in a case with trisomy detectable in only a minority of fetal heart cells.^83^ In our cases, CHD could arise if trisomy-16 cells persist at low levels in embryonic lineages contributing to cardiac development, even when blood lymphocytes show apparent reversion to diploidy and therefore only maternal UPD16.

Despite the strengths of our study, several limitations warrant consideration. First, our cohort is underrepresented for non-European ancestries, which may limit the generalizability of our findings across populations. Because genetic diversity could influence both the prevalence and haplotype background of UPD, the distribution of UPD types and relationships to CHD may differ in other ancestry groups. Second, although we analyzed the largest parent-proband sequencing-based CHD cohort to date, UPD events remain rare. This limited sample size reduces power to evaluate the full spectrum of UPD in CHD and to detect associations for less common UPD classes or rare chromosomal events. In addition, our approach has limited sensitivity for very small UPD events, which may lead to under detection of subtle segmental UPDs. Third, differences between the case and control cohorts introduce potential bias. UPD in the 23andMe cohort was detected using SNP microarray, whereas UPD in our case cohort was detected using whole exome or genome sequencing; differences in genomic coverage and breakpoint resolution could cause segmental UPD event detection in one cohort but not in the other. To improve comparability, we matched study design where possible. For prevalence estimates, we compared case trios with control trios, consistent with the published approach for deriving the ~0.05% UPD prevalence in 23andMe.^13^ In addition, when estimating per-chromosome UPD burden, we restricted analyses to whole-chromosome UPD, which is unlikely to be missed across platforms. Finally, the 23andMe cohort represents a general population sample rather than a rigorously phenotyped “healthy” control set; although we confirmed the absence of reported cardiac phenotypes among controls, participants may have other traits or diagnoses that could be associated with UPD.

In conclusion, this study provides the first systematic assessment of the full spectrum of UPD in CHD, addressing two central questions: how UPD arises, and how it contributes to disease. We show UPD susceptibility is shaped by age-related and heritable influences on the fidelity of chromosome segregation, and potentially by their combined effects. By integrating cohort-scale UPD detection, long-read sequencing, and *in vitro*/*in vivo* functional modeling, we refine the genetic architecture of CHD and identify UPD as an underrecognized mechanism that can unmask damaging recessive alleles. Candidate genes identified through UPD analysis may also help resolve previously unexplained cases by directing attention to recessive variants that were missed during discovery analyses because of limited statistical power. These findings support incorporating UPD analysis into genetic evaluation frameworks for CHD, particularly in cases suggestive of recessive inheritance. Additionally, we emphasize the importance of considering maternal-age–associated genetic risks and support the value of appropriate prenatal counseling and postnatal genetic evaluation in selected cases. More broadly, the same UPD-based mechanisms are likely relevant to other recessive conditions, including metabolic disorders such as lysosomal storage diseases, where homozygosity for a pathogenic allele can have profound clinical consequences.

## ONLINE METHODS

### Patient cohorts

Subjects with CHD were recruited through the Congenital Heart Disease Genetic Network Study of the Pediatric Cardiac Genomics Consortium (CHD GENES: ClinicalTrials.gov identifier NCT01196182). Written informed consent was obtained from all participants or their parents/guardians in accordance with institutional review board-approved protocols.

Individuals were enrolled based on a confirmed clinical diagnosis of structural CHD. Cardiac diagnoses were verified through comprehensive reviews of imaging and operative reports and coded using Fyler codes derived from the International Pediatric and Congenital Cardiac Code (http://www.ipccc.net/). At enrollment, all participants underwent standardized assessments that included detailed interviews capturing maternal, paternal, and birth history, as well as documentation of prior genetic evaluations.

A thorough medical record review was conducted for each proband to collect anthropometric data (height and weight), document the presence or absence of extracardiac malformations, summarize available genetic testing and results, and record any established clinical genetic diagnoses. For probands under one year of age, consultations with non-cardiology specialties obtained during routine clinical care were recorded. For probands older than one year, parents were asked about developmental delay and the use of educational or therapeutic support services. A three-generation pedigree was obtained for every family enrolled in the study.

### Cardiac phenotyping

Cardiac phenotypes were classified into five major categories based on the major structural lesion: conotruncal defects (CTD), D-transposition of the great arteries (D-TGA), heterotaxy (HTX), left ventricular outflow tract obstruction (LVO), and Other Category. The CTD group included TOF, double-outlet right ventricle (DORV), truncus arteriosus, membranous ventricular septal defects (VSD), and aortic arch abnormalities. The LVO group comprised hypoplastic left heart syndrome (HLHS), coarctation of the aorta (CoA), and aortic stenosis/bicuspid aortic valve (AS/BAV). HTX syndromes were defined by abnormal situs, such as dextrocardia, left or right isomerism (LAI, RAI) as the major malformation, and may include other defects such as L-transposition of the great arteries (L-TGA), AVC, anomalous pulmonary venous drainage (TAPVR, PAPVR), and double outlet right ventricle. Isomerism of other organs was not considered as a separate extra-cardiac malformation in this study. The “Other Category” included pulmonary valve abnormalities, anomalous pulmonary venous drainage, atrial septal defects (ASD), AVC, double inlet left ventricle (DILV), and tricuspid valve atresia (TA). Any non-acquired structural anomaly outside the heart was classified as an extracardiac malformation.

### Exome/genome sequencing experiment, sequencing analysis,

Exome sequencing was performed at the Yale Center for Genome Analysis following the same protocol as previously described.^7^ Genomic DNA from venous blood or saliva was captured using the Nimblegen v.2 exome capture reagent (Roche), Nimblegen SeqxCap EZ MedExome Target Enrichment Kit (Roche), or the xGen target capture kit (IDT) followed by Illumina sequencing as previously described.^7^

Genome sequencing was conducted at the Baylor College of Medicine Genomic and RNA Profiling Core (n = 297), the New York Genome Center (NYGC) Genomic Research Services (n = 25), the Broad Institute for Genomic Services (n = 596), and the University of Washington (n = 5), following the same protocol as previously described.^84^ Genomic DNA from venous blood or saliva was prepared for sequencing using a PCR-free library preparation or SK2-IES library preparation. All samples were sequenced on an Illumina Hi-Seq X Ten system with 150-bp paired reads to a median depth of >30× per individual. Raw sequence reads were aligned to the human reference genome GRCh38 with bwa mem.^85^ GATK Best Practices workflows with duplication marking, indel realignment, and base quality recalibration were used to process aligned reads.^86,87^ CRAM alignments were screened for contamination using VerifyBamID2, with Freemix scores of 0.05 or greater being rejected.^88^

### Detection of UPD events

A total of 3,740 parent-proband trios from the PCGC cohort that were available as of December 2022 were selected for UPD analysis. The docker image (https://hub.docker.com/r/jinlab/triomix/) was utilized to download and execute TrioMix-UPD (https://github.com/NahyunKong/triomix-upd), a specialized version of TrioMix^21^ designed to detect UPD from trio-based sequencing data, supporting WES, WGS, and long-read sequencing. This tool identified UPD events by categorizing SNVs into two groups based on the genotypes of the parents (**Supplementary Figure 7**). Any variants that are homozygous reference genotype (0/0) in one of the parents and homozygous alternative genotype (1/1) in the other parent is defined as Group A. The child’s expected VAF for Group A is 0.5 based on Mendelian inheritance. Alternatively, variants that are homozygous reference genotype (0/0) in one of the parents and heterozygous genotype in the other parent (0/1) are classified as Group B. The expected VAF for the child in Group B varies, being either 0 or 0.5, with a 50% probability for each. Violations of these expected VAFs indicate the presence of UPD. Specific UPD subtypes produce distinct patterns within each group: in Group A, strong deviations toward ~0 or ~1 suggest UPD, while in Group B, a child VAF pattern of 0 or 1 indicates isodisomy, and a pattern of 0 or 0.5 indicates heterodisomy. Segmentation was performed per SNP group using CBS (PSCBS R package)^89^, with outlier removal and large gap detection applied prior to fitting; segments supported by fewer than 10 variants were excluded. Candidate UPD regions were additionally filtered by read depth, removing segments with median depth below 60% of the autosomal genome-wide median to exclude copy-number losses mimicking UPD. Genomic segments longer than 100 kb that consistently matched these patterns were identified as UPDs and outputted as a BED file. All identified UPD events were manually verified using the Integrative Genomics Viewer.^90^

#### Control cohort:

The control group consists of genome-wide array data from 916,712 parent-child duos within the 23andMe database,^13^ as previously described in published research.^91,92^ UPD detection in the 23andMe cohort was performed using the GARLIC tool to identify runs of homozygosity (ROH) across the genome.^93^ A total of 205 UPD events were identified from the 916,712 parent-child duos. Case-control burden tests were then conducted to compare the frequency of UPD in our CHD cases with that in the 23andMe controls.

### Kinship analysis

We assessed the relationship between each proband and their parents by performing pairwise identity-by-descent estimation using PLINK.^94^ Across all trios, the proportion of identify by descent sharing between probands and their parents consistently fell within the expected range of 45–55%.

### Principal component analysis

We inferred the ancestry of each sample using the EIGENSTRAT^95^ software, based on tag SNPs from study cases, controls, and HapMap reference populations, following previously described methods.^7^

### Burden test in chromosome segregation genes

We analyzed rare LoF and missense variants in 12 UPD trios and 3,728 non-UPD trios from the PCGC cohort. GATK HaplotypeCaller was used to generate GVCF files, which were combined into a multisample exome or whole-genome VCF using the GATK tools GenomicsDBImport and GenotypeGVCFs.^96^ In the case of exomes, GVCFs were generated at base pair resolution. Variant Quality Score Recalibration (VQSR) was used for variant-level filtration, with the truth sensitivity threshold set to 99.50 for both SNVs and indels. Variants were annotated using ANNOVAR-based functional annotations including gene consequence, gnomAD population frequencies (v2.0.1)^33^ and BRAVO.^34^ Only variants with alternate allele genotypes, sequencing depth ≥8, and maximum population allele frequency (AF) <0.001 were retained for burden analyses. Variants were classified as LoF, including stop gain, stoploss, frameshift insertion/deletion/substitution, start loss, and splice-site variants, or missense variants.

Gene-level and pathway-level burden analyses were performed separately in UPD probands and parents of UPD probands. We curated biologically informed gene sets involved in meiotic chromosome segregation and genome stability, including synaptonemal complex, meiotic recombination, cohesin complex, spindle assembly checkpoint, centrosome biogenesis, and spindle positioning pathways (**Supplementary Table 3**). For each gene or pathway, carrier frequencies in cases and controls were compared using two-sided Fisher’s exact test. Odds ratios (ORs), 95% confidence intervals (CIs), and p-values were calculated, followed by Bonferroni correction for multiple testing. At the gene level, each individual was counted once per gene regardless of the number of variants carried. At the pathway level, each individual was counted once per biological pathway.

### Identification of rare damaging SNVs in isodisomy regions

SNVs and small indels were called with GATK Haplotype Caller and annotated using ANNOVAR^97^. The CADD v.1.6 algorithm was used to predict the deleteriousness of the missense variants.^98^ Variants were filtered for rare (MAF ≤ 0.01 across all samples in gnomAD v2.0.1^33^ and BRAVO.^34^ Homozygous variants within isodisomy regions were selected based on high-quality sequencing metrics, defined as a minimum of 8 total reads and genotype quality (GQ) ≥ 20 for the proband, and at least 4 total reads with GQ ≥ 10 for each parent. These variants were required to be homozygous alternate (homozygous alt) in the proband, heterozygous in the UPD-inheriting parent, and homozygous reference (homo ref) in the other parent. LoF variants (nonsense, canonical splice-site, frameshift indels, and start loss) or missense variants with CADD scores were further classified according to ACMG/AMP guidelines.^38^ All variants reported in the present work were manually inspected and confirmed using the Integrative Genomics Viewer (IGV).

### PacBio circular consensus (CCS or HiFi) sequencing

High-fidelity whole-genome sequencing was performed on the PacBio Revio platform at the Broad Institute for four UPD trios (1–02917, 1–03820, 1–06216, and 1–14785). High-molecular-weight genomic DNA was first purified using the MagAttract HMW DNA Kit (Qiagen). At least 2 μg of high molecular weight genomic DNA (>50% of fragments ≥ 40 kb) was sheared to ~15 kb using the Megaruptor 3 (B06010003), followed by DNA repair and ligation of PacBio adaptors using the SMRTbell Prep Kit v3.0 (102–141-700). Each library was subsequently size selected for 10 kb ± 20% using the PippinHT with 0.75% agarose cassettes (Sage Science). After quantification with the Lunatic (Unchained Labs), libraries were diluted to 250 pM per single molecule, real-time (SMRT) cell, hybridized with PacBio standard sequencing primer, and bound with SMRT sequencing polymerase using the Revio polymerase kit (102–739-100). CCS sequencing was performed on the Revio instrument using 25M SMRT Cells (102–202-200) and Revio SPRQ Sequencing Plate (103–504-900 or 102–587-400), with a 2-hour pre-extension time and 30-hour movie time per SMRT cell. Primary data review including quality filtering, base calling, and demultiplexing are performed via onboard processors.

Error correction for reads generated in CCS mode was performed on-board with the vendor’s ccs software (https://github.com/PacificBiosciences/pbccs) and settings --all --subread-fallback --num-threads 232 –streamed <movie_name>.consensusreadset.xml --bam <movie_name>.reads.bam. The resulting reads, including those that failed error correction and packaged into a single BAM file for further analysis. Should a run contain individually barcoded libraries, on board demultiplexing will produce a single unique BAM file for each index present. The average HiFi read length was 15 kb with 22× depth for the four UPD trios. Per-sample depth and additional metrics are reported in **Supplementary Figure 8**.

### Long-read SV analysis and annotation, and assessment of methylation disruption

Reads were aligned to GRCh38 using pbmm2^99^ to generate mapped BAMs. Structural variants were called with Sniffles2 (v2.0.3)^100^ and, for the UPD isodisomy regions, we retained structural variants that were homozygous in the proband and heterozygous in the UPD-contributing parent but absent in the other parent using bcftools (v1.21)^101^ and Truvari (v4.0.0).^102^ Population filtering was performed by harmonizing long-read structural variant resources with needLR (v3.4), followed by removal of structural variants with Allele_Freq_ALL > 0.01 from nanopore sequencing of 450 samples from the 1000 Genomes Project.^39^ Remaining structural variants were functionally annotated with AnnotSV (v3.4.4)^40^ with an option “-benignAF 0.01”, and we excluded events classified as benign/likely benign from their ACMG classification or not overlapping gene or other regulatory elements (**Supplementary Figure 9**). Lastly, each SV were independently confirmed by IGV visualization (**Supplementary Table 6**).

For methylation analysis, phased BAMs were generated by calling small variants using DeepVariant^103^, and haplotype phased with HiPhase^104^. We then identified regional methylation outliers using METAFORA^22^, which (i) computes a cohort-weighted population methylation profile at each CpG (depth-weighted, with depth capped to limit inflation at very high coverage), (ii) transforms per-CpG deviation from the population profile into a depth-aware deviance/z-score signal, (iii) performs genome-wide segmentation of this signal to nominate contiguous candidate regions containing at least a minimum number of CpGs, and (iv) aggregates CpG calls within each candidate region to estimate region-level methylation and test outlier status using cohort z-scores on batch-/covariate-corrected M-values (M-values are the log_2_ ratio of methylated to unmethylated CpG read counts). Candidate regions were retained if they exceeded both an effect-size threshold (delta= 30% relative to the population median) and a z-score = 2 threshold and overlapping outlier regions were merged and jointly re-scored across samples. Each proband was compared against controls, including their own parents and other parents from the PCGC cohort, using PacBio whole-blood sequencing data generated at the same institute (90 parents, 45 female and 45 males; data QC available at **Supplementary Figure 8**).

### Zebrafish Lines, Husbandry, and Genotyping

The work involving Zebrafish (*Danio rerio*) and the experimental procedures in this study were approved by the Washington University in St. Louis School of Medicine Institutional Animal Care and Use Committee (IACUC).

The transgenic line *Tg(7xTCF-Xla.Sia:nlsmCherry)*^*ia5*^ was used to analyze Wnt activity during development.^55^ We utilized the zebrafish mutant lines *piezo1*^*sa12608*^ *(ZL9041.03)* and *piezo2a.1*^*sa12414*^ *(ZL8936.10)*^105^, and crossed them with the *Tg(tagln:eGFP*^*p151*^*); (kdrl:mCherry-CAAX*^*y171*^*)* to visualize the smooth muscle (*tagln*-positive) cells and the endothelial (*kdrl*-positive) cells of the endocardium.^106–108^ Genotyping of the *piezo1* and *piezo2a.1* fish lines was performed using the Kompetitive Allele Specific PCR assay (KASP). The KASP assay for *piezo1* and *piezo2a.1* was synthesized by LGC Biosearch Technologies (project number 1166.002). For genotyping the adult zebrafish, the fish were anesthetized with 100mg/mL of MS-222 (Syndel USA, Cat. #: NC0872873) to obtain a small piece of the tail fin for gDNA extraction. gDNA extraction was carried out in 0.2mL 8-Strip PCR tubes (Alkali Scientific, Cat. #: PC7061), and gDNA extraction buffer (10mM Tris-HCl pH: 8.2, 200mM NaCl, 0.5% SDS, and 200μg/mL of Proteinase K) at room temperature. The KASP reaction was prepared following the manufacturer’s recommendations in 1x master mix (LGC Biosearch Technologies, Cat. #: KBS-1050), and the subsequent allele amplification was performed using a QuantStudio 3 Real-Time PCR System (ThermoFisher Scientific, Cat. #: A28567).

### Human Pro42Leu *PIEZO1* and Pro29Ser *MESD* plasmids

Plasmids containing the *Homo sapiens PIEZO1* sequence (Cat. #: OHu23112D) and *MESD* sequence (Cat. #: OHu06853D) were purchased from GenScript. To generate the human *PIEZO1* missense variant, we introduced a proline 42 to leucine G>A mutation in the plasmid using a combination of the Q5 Site-Directed Mutagenesis Kit (New England Biolabs, Cat. #: E0554S) and the NEBuilder^®^ HiFi DNA Assembly Cloning Kit (New England Biolabs, Cat. #: E5520S). Whole Plasmid Sequencing was performed by Plasmidsaurus using Oxford Nanopore Technology with custom analysis and annotation to confirm the edited plasmid. The same pipeline was done for *MESD* to introduce the proline 29 to serine G>A mutation. The PIEZO1-GCaMP6f WT plasmid was generated and validated by the M, Pathak Lab. The proline 42 to leucine G>A mutation was induced following the protocol outlined above.

### *PIEZO1* Plasmid injection and CRISPR/Cas12a injection

Adult zebrafish *piezo1*^*sa12608/+*^*; piezo2a.1*^*sa12414/sa12414*^ zebrafish mutants carrying the *Tg(tagln:eGFP*^*p151*^*); (kdrl:mCherry-CAAX*^*y171*^) transgenes were in-crossed.^46^ Eggs were collected and injected at the one-cell stage with 2 nL of 25 μg stock plasmid— either the WT human *PIEZO1* plasmid or the mutated plasmid (*PIEZO1*^*Pro42Leu*^)— and 50 ng of Tol2 mRNA. For the vehicle injection control, we utilize the same injection solution minus the plasmids. The embryos were incubated at 28°C for 72 hours before imaging analysis. For imaging, living embryos were vertically mounted in 1% low melt agarose (IBI, Cat. #: 18E4002) to obtain ventral images of the heart, outflow tract, and aorta using a W1 spinning disk confocal microscope at 10x or 20x magnification. Post-imaging, the embryos were individually collected and stored at −20°C for gDNA extraction and genotyping.

CRISPR guide RNAs and Cas12a were injected following the same basic protocol, injecting the CRISPR guides in 2 nL of 25–100 ug stock sgRNA with 10 ug stock Cas12a (Alt-R A.s. Cas12a (Cpf1) Ultra; IDT, Cat. #10001272) following the protocol outlined in previous publications.^109^ In all cases, Cas12a injected with no guide RNA was used as a control.

CRISPR/Cas12a targets used for this study:

Two guides per gene were tested, both showing the same phenotypic effects (**Supplementary Figure 6**). The data from 1 guide site is presented in the manuscript and indicated below. Guides were selected using ChopChop and the (danRer11/GRCz11) *Danio rerio* genome.^110^

*mesd* sgRNA1: TTCTCGTTAGGTTTGTGGTGGGCTCTAA

*mesd* sgRNA2: TTCTCTGTGGGGTTCCCTGACACTGATG (guide used for data shown)

*glyr1* sgRNA1: TTCTGCAGGTATACAAGAGAGCAAAAGC (guide used for data shown)

*glyr1* sgRNA2: TTCACCACATGCTTTCTTACCTCGTTTG

### PIEZO1 puncta analysis

Images were analyzed using an automated particle-counting workflow in ImageJ/Fiji. Images converted to 8-bit grayscale. To isolate the objects of interest (“PIEZO1 puncta”), a threshold was applied using Image > Adjust > Threshold, and the sliders were adjusted until the dots were highlighted while the background remained unselected, and then uniformly applied to all images. The thresholded image was then converted to a binary image. When adjacent dots appeared to touch, they were separated using the watershed function. Particle analysis was performed on selected ROIs and the option Show: Outlines was selected to visualize detected particles. The percentage area of the RO1 that the PIEZO1 puncta encompassed was calculated automatically and reported in the corresponding figure.

### *PIEZO1* Pressure Patch-Clamp experiments

PIEZO1 knocked out HEK293T cells (gifted from Ardèm Patapoutian, Scripps Research) were grown in a 24 well plate and transfected with 1 μg of PIEZO1WT_GCaMP or PIEZO1Pro42Leu_GCaMP plasmid. Transfected cells were re-seeded on glass coverslips coated with Matrigel (Corning) at a 1/50 dilution one day after transfection and used in patch clamp experiments two days after transfection. Patch pipettes were pulled from G150F borosilicate capillaries (Warner Instruments) to a resistance of 3–4 MΩ using a P-97 puller (Sutter Instrument) and heat-polished using a Microforge-MF2 (Narishige). PIEZO1 currents were activated by mechanical poking of transfected cells using a blunt glass probe attached to a piezoelectric actuator (Physik Instrumente). Pipettes were filled with 140 mM KCl, 10 HEPES, 10 mM TEA and 2 mM EGTA (pH 7.4 with NaOH). HBSS (GIBCO) was used as bath solution.

### *PIEZO1* GCaMP experiment

HUVECs plated on cover slips were transfected with 500 ng of WT or *PIEZO1*^*Pro42Leu*^ plasmid using Lipofectamine 2000. 24 hours after transfection, the cells were fixed in 4% PFA, and then immunostained with an anti-GFP antibody (Invitrogen; #MAB-15256) to maintain coordinated timing of GCaMP6f expression between all conditions. STED stable secondary antibody (*abberior* Star Orange, goat anti-mouse #STORANGE-1001–550UG) was used and 2D super resolution STED imaging on single imaging planes done using a 2D STEDYCON microscope (*abberior*). GFP antibody was used to amplify the endogenous GCaMP6f signal following fixation, to assess dispersion of the GCaMP6f fused Piezo1 WT versus Pro42Leu channels.

### Cell culture and immunostaining

Human umbilical vein endothelial cells (HUVECs, Lonza) were cultured in growth media of M199 (#11150059, Gibco), 20% fetal bovine serum (FBS, Gibco), 0.05 g heparin sodium salt (#H3393, Sigma-Aldrich), and 15 mg ECGS (#02–102, Sigma-Aldrich). Cells were cultured on gelatin-coated plates at 37°C, 5% CO_2_. HUVECs were used from passages 2–6.

For immunofluorescence staining, cells were fixed in 4% paraformaldehyde for 10 minutes and permeabilized with 0.1% Triton X-100 in PBS for 30 minutes, followed by blocking with Tris-glycine for 30 minutes. Primary antibody incubation was performed at RT for 1 hour. Secondary antibody incubation was performed for 1h at room temperature at 1:2000 dilution using highly cross-adsorbed Alexa Fluour secondary antibodies. Nuclei were stained with Hoechst 34580 (#H21486, Invitrogen) for 15 minutes at room temperature. Imaging was performed using a W1 spinning disk confocal microscope (40x objective). Image analysis was performed in Fiji. Primary antibodies: T. LRP6 (#MAB1506, RD Systems); p-LRP6 Ser1490 (#2568, Cell Signaling).

### Statistical analysis for graphical data

Statistical analyses were performed using GraphPad Prism 11 (GraphPad Software, Inc). Data normality was determined using the D’Agostino-Pearson test. Data with normal distribution were analyzed using unpaired Student t tests (data with 2 groups), ordinary one-way ANOVAs (data with >2 groups), and two-way ANOVAs (data with multiple variables). Data with non-normal distribution were analyzed using Kruskal-Wallis tests (data with >2 groups). Post hoc multiple comparison tests were selected based on the recommendation of GraphPad Prism 11. Significance was determined by a p-value of ≤0.05. Statistical analyses, post hoc tests, and p-values are all described in corresponding figures and figure legends.

## Supplementary Material

Supplementary Files

This is a list of supplementary files associated with this preprint. Click to download.

- SupplementaryTables05302026.xlsx
- SUPPLEMENTARYINFORMATION.pdf

## Figures and Tables

**Table 1.** Twelve UPD events identified in 3,740 PCGC trios. The proband identifier, UPD subtype, cardiac diagnosis, and any reported syndromic diagnosis were listed.

Proband ID	UPD type	Diagnosis	Syndrome
1–00660	upd(4)mat heterodisomy	Tetralogy of Fallot	N/A
1–08240	upd(4)mat mixed	Atrial septal defect, secundum | Bicommissural aortic valve | Coarctation off the aorta | Left superior vena cava connecting to the coronary sinus | Ventricular septal defect, membranous | Ventricular septal defect, single	N/A
1–03573	upd(8)mat segmental isodisomy	Atrial septal defect, secundum | Bicommissural aortic valve | Coarctation of the aorta | Transitional common AV canal | Tubular hypoplasia of the aortic arch | Ventricular septal defect, muscular	N/A
1–04975	upd(9)mat heterodisomy	Left superior vena cava connecting to the coronary sinus | Pulmonary atresia congenital | Right superior vena cava connecting to the right atrium | Right ventricular origin of subaortic conus | Ventricular septal defect	N/A
1–15783	upd(12)mat isodisomy	Agenesis of kidneys | Atrial septal defect | Patent ductus arteriosus | Tetralogy of Fallot	Trisomy X
1–00180	upd(14)mat mixed	Aortic stenosis, subvalvar, discrete, membraneous | Bicommissural aortic valve | Coarctation of the aorta, simple (isolated) | Imperforate anus | Pulmonary arterial disease | Ventricular septal defect, membranous	N/A
1–03820	upd(15)mat mixed	Cleft mitral valve | Left superior vena cava connecting to the coronary sinus | Ostium primum atrial septal defect, balanced | Ventricular septal defect, multiple | Ventricular septal defect, muscular, trabecular, apical	Prader-Willi syndrome
1–02917	upd(16)mat mixed	Left superior vena cava connecting to the coronary sinus | Tetralogy of Fallot with pulmonary atresia	N/A
1–06216	upd(16)mat mixed	Atrial septal defect, secundum	N/A
1–14523	upd(16)mat mixed	Coarctation of the aorta	N/A
1–14785	upd(16)mat mixed	Truncus arteriosus type I (main pulmonary artery present)	N/A
1–15162	upd(19)pat segmental isodisomy	Tricuspid atresia	N/A

PCGC: Pediatric Cardiac Genomics Consortium.

**Table 2.** Rare potentially damaging homozygous variants within UPD isodisomy regions identified by short-read sequencing. The table lists the proband identifier, gene harboring the homozygous variant, amino acid change, variant consequence/type, gnomAD minor allele frequency (v2.0.1), CADD score (v.1.6), and ACMG/AMP classification.

Proband ID	Gene	Protein effect	Consequence	gnomAD MAF	CADD	Known Gene–Disease Associations	Known CHD gene	ACMG/AMP criteria	Variant Classification
1–14523	*PIEZO1*	p.Pro42Leu	nonsynonymous	5.30E-06	26.7	AD dehydrated hereditary stomatocytosis with or without pseudohyperkalemia and/or perinatal edema (OMIM 194380); AR lymphatic malformation 6 (OMIM#616843)	No	PM2_supporting, PP3	VUS
	*UBN1*	p.Gln381Lys	nonsynonymous	1.20E-06	16.5	No	No	N/A	GUS
	*CACNA1H*	p.Arg1597Gln	nonsynonymous	1.80E-04	25.2	AD hyperaldosteronism, familial, type IV (OMIM 617027)	No	BS1, PP3	VUS
1–15783	*MYO1A*	p.Tyr740Ter	stop gain	9.90E-05	34	No	No	N/A	GUS
	*SLC6A13*	p.Ser397Thr	nonsynonymous	1.60E-05	10.6	No	No	N/A	GUS
	*DENND5B*	p.Ile624Val	nonsynonymous	9.20E-05	23	No	No	N/A	GUS
	*CKAP4*	p.Arg454Cys	nonsynonymous	3.30E-05	26.5	No	No	N/A	GUS
1–06216	*DNAAF1*	p.Met202Val	nonsynonymous	1.58E-04	22.8	AR ciliary dyskinesia, primary, 13 (OMIM 613193)	Yes (heart laterality defects, see PMID: 29228333)	PM2_supporting	VUS
	*C16ORF89*	p.Ser74Thr	nonsynonymous	2.40E-04	14.9	No	No	N/A	GUS
	*RIPOR1*	p.Ser785Tyr	nonsynonymous	7.20E-06	18.4	No	No	N/A	GUS
	*WWP2*	p.Met757Val	nonsynonymous	6.20E-06	9.5	No	No	N/A	GUS
	*ZDHHC1*	p.Gly330Trp	nonsynonymous	2.50E-05	22.1	No	No	N/A	GUS
	*ATXN1L*	p.Thr109Met	nonsynonymous	1.40E-05	23.4	No	No	N/A	GUS
1–03820	*MESD*	p.Pro29Ser	nonsynonymous	8.75E-05	11.82	AR osteogenesis imperfecta, type XX (OMIM 618644)	No	PM2_supporting	VUS
1–02917	*CASKIN1*	p.Arg619Cys	nonsynonymous	3.40E-05	27.1	No	No	N/A	GUS

AA, amino acid; gnomAD, Genome Aggregation Database; CADD, Combined Annotation Dependent Depletion; ACMG/AMP, American College of Medical Genetics and Genomics/Association for Molecular Pathology.

**Table 3.** Methylation outliers identified in trios with PacBio HiFi sequencing. All long-read sequencing data were generated at the Broad Institute to minimize batch effects. Methylation outlier regions were identified using METAFORA by comparing each of the four UPD probands to a reference set of 90 unaffected parents from the PCGC cohort. Regions with an absolute z-score > 2 are reported, along with proband ID, chromosome, genomic coordinates (Start/End), number of CpG sites in the region, the mean population deviation score across the region (Mean Deviation), the median methylation level in the reference population (Pop Median), the absolute methylation difference relative to the population median (Delta), z-score, any known disease-associated imprinted gene within the region, and predicted enhancers based on the Activity-by-Contact model (ABC Enhancer Name).

Proband ID	Chr	Start	End	# CpG	Mean Deviation	Pop Median	Delta	Z-score	Known imprinted gene associated with disease	ABC Enhancer Name
1–02917	16	3431602	3435848	54	3.90	0.64	0.33	2.53	N/A	*ZNF597,NAA60,ZNF174,ZSCAN32*
	16	3442827	3444461	76	−4.32	0.51	−0.49	−3.32	NA	*ZNF597*
	16	29317783	29318549	20	−2.32	0.87	−0.31	−2.08	NA	*SNX29P2*
	16	60647175	60647821	32	7.37	0.20	0.76	2.07	NA	NA
	16	65463230	65466680	41	4.58	0.43	0.48	2.08	NA	NA
	16	83443113	83444894	22	3.60	0.64	0.32	2.51	NA	NA
1–03820	15	24477570	24478450	41	2.06	0.26	0.27	2.05	NA	NA
	15	24954396	24956827	118	4.41	0.53	0.42	3.25	*SNRPN/SNURF*	*SNURF,PWAR5,ATP10A,UBE3A,WHAMMP3,NDN,MKRN3,MAGEL2,HERC2P2,SNRPN,PWAR1,GABRA5,SNORD115–15,SNORD115–21,NPAP1,GOLGA6L2,PWRN1*
	15	94242933	94245592	91	6.49	0.29	0.69	2.56	NA	NA
	15	97483313	97484625	30	6.14	0.38	0.59	2.82	NA	NA
1–14785	16	3442827	3444461	76	−4.76	0.51	−0.49	−2.10	NA	*ZNF597*

## Data Availability

All phenotypic and sequencing data generated by the Pediatric Cardiac Genomics Consortium are deposited under dbGaP accession number phs001194 and can be accessed after approval by the NHLBI’s Data Access Committee. Data are physically stored in NHLBI’s BioData Catalyst ecosystem (https://biodatacatalyst.nhlbi.nih.gov/).

## CODE AVAILABILITY

The software utilized in this study is available at the following web addresses: TrioMix-UPD is available at https://github.com/NahyunKong/triomix-upd
